# Dr. Julius L. Salinger

**Published:** 1912-05

**Authors:** 


Dr. Julius L. Salinger, of Philadelphia, a graduate of Jefferson College, 1886, later a teacher; died of angina pectoris March 24, 1912, while testifying in court. He was a neurologist of wide reputation. His untimely death, at the age of 46, cuts short a career that, for the average man would be an attainment but which for him, was but a promise.

